# Application of the advanced lung cancer inflammation index for patients with coronavirus disease 2019 pneumonia: Combined risk prediction model with advanced lung cancer inflammation index, computed tomography and chest radiograph

**DOI:** 10.3892/etm.2022.11315

**Published:** 2022-04-12

**Authors:** Akitoshi Inoue, Hiroaki Takahashi, Tatsuya Ibe, Hisashi Ishii, Yuhei Kurata, Yoshikazu Ishizuka, Bolorkhand Batsaikhan, Yoichiro Hamamoto

**Affiliations:** 1 Department of Radiology, Shiga University of Medical Science Seta, Otsu, Shiga 520-2192, Japan; 2 Department of Radiology, Mayo Clinic, Rochester, MN 55905, USA; 3 Department of Plumonary Medicine, National Hospital Organization Nishisaitama-Chuo National Hospital, Tokorozawa, Saitama 359-1151, Japan; 4 Department of Radiology, National Hospital Organization Nishisaitama-Chuo National Hospital, Tokorozawa, Saitama 359-1151, Japan; 5 Department of Radiological Science, Graduate School of Human Health Sciences, Tokyo Metropolitan University, Tokyo 116-8551, Japan

**Keywords:** COVID-19, lung cancer, inflammation, disease exacerbation, computed tomography, chest radiograph

## Abstract

The purpose of the present study was to evaluate the feasibility of applying the advanced lung cancer inflammation index (ALI) in patients with coronavirus disease 2019 (COVID-19) and to establish a combined ALI and radiologic risk prediction model for disease exacerbation. The present study included patients diagnosed with COVID-19 infection in our single institution from March to October 2020. Patients without clinical information and/or chest computed tomography (CT) upon admission were excluded. A radiologist assessed the CT severity score and abnormality on chest radiograph. The combined ALI and radiologic risk prediction model was developed via random forest classification. Among 79 patients (age, 43±19 years; male/female, 45:34), 72 experienced improvement and seven patients experienced exacerbation after admission. Significant differences were observed between the improved and exacerbated groups in the ALI (median, 47.6 vs. 13.2; P=0.011), frequency of chest radiograph abnormality (24.7 vs. 83.3%; P<0.001), and chest CT score (CCTS; median, 1 vs. 9; P<0.001). For the accuracy of predicting exacerbation, the receiver-operating characteristic curve analysis demonstrated an area under the curve of 0.79 and 0.92 for the ALI and CCTS, respectively. The combined ALI and radiologic risk prediction model had a sensitivity of 1.00 and a specificity of 0.81. Overall, ALI alone and CCTS alone modestly predicted the exacerbation of COVID-19, and the combined ALI and radiologic risk prediction model exhibited decent sensitivity and specificity.

## Introduction

After ~2 years of coronavirus disease 2019 (COVID-19) pandemic caused by severe acute respiratory syndrome coronavirus 2 which was first identified in 2019(1), most countries have faced multiple surges of newly diagnosed patients with COVID-19(2). Patients with COVID-19 are treated with existing and newly developed therapeutic approaches according to the severity of the disease (3,4). Most patients without symptoms are treated in primary care facilities with supportive care, including oxygen inhalation and vital sign monitoring. Even symptomatic patients whose status requires inpatient care are sometimes forced to stay at home due to the limitations of medical resources (5). An increased risk of cardiac arrest at home has been reported among COVID-19 patients (6). Exacerbation of symptoms is common in inpatient care (7), and 15-30% of hospitalized patients develop acute respiratory distress syndrome (8). Therefore, the establishment of practical and concise criteria to triage COVID-19 patients is necessary to prepare for another wave of the pandemic surge when medical resources are limited.

Specifically, in the field of radiology, there is a high demand for diagnostic imaging, including chest radiography and computed tomography (CT), to assess the severity and future risk of exacerbation in COVID-19 patients (9-11). However, the resources available for diagnostic imaging are limited, especially for at home medical care or in local community hospitals; therefore, unnecessary imaging studies should be avoided. Thus, the establishments of practical triage systems will help reduce the burden of radiologic examinations under pandemic surges.

The advanced lung cancer inflammation index (ALI) was initially developed as a prognostic indicator for metastatic non-small cell lung cancer (12). ALI is currently used for other types of neoplasm, including colorectal cancer, lymphoma, and pancreatic cancer (13-16). as well as for non-neoplasmic diseases such as Crohn's disease (17). ALI is calculated using body mass index (BMI), serum albumin level, neutrophil count, and lymphocyte count (The forumula is described in Material and Method section), which are measured and readily available at primary care facilities (18). Because the variables used in the ALI calculation are known to be correlated with the severity of COVID-19 patients, the ALI is presumed to be related to the prognosis of these patients. For instance, patients with both hypoalbuminemia and lymphopenia have a high risk of severe COVID-19(19), and obesity increases the risk of hospitalization, intensive care unit admission, and death (20,21).

This study aimed to assess the feasibility of applying the ALI in COVID-19 patients; evaluate the correlation among the ALI, imaging studies (radiography and CT), and prognosis; and establish concise triage criteria to mitigate workload in the radiology service under a pandemic surge.

## Materials and methods

### Patients

This retrospective study was approved by the IRB of National Hospital Organization Nishisaitama-Chuo National Hospital (IRB no. 2020-18), and written informed consent was waived. We enrolled patients admitted to National Hospital Organization Nishisaitama-Chuo National Hospital from March to October 2020 due to diagnosis of COVID-19 infection *via* polymerase chain reaction test of respiratory tract specimen. Patients without clinical information and/or chest CT upon admission were excluded (13 patients). A total of 79 patients (age: 43±19 years, male/female: 45:34) were enrolled.

### Clinical parameters

A pulmonologist (Y.H.) reviewed the patients' medical records to abstract age, sex, body weight (kg), height (cm), BMI (kg/m^2^), white blood cell (WBC) count (/µl), neutrophil/lymphocyte ratio (NLR), albumin level (g/dl), clinical severity at admission, and clinical course. Clinical severity upon admission was classified into two categories: mild, percutaneous oxygen saturation (SpO_2_) ≥93%; severe, SpO_2_ <93% or requiring oxygen inhalation. One of our authors (Y.I.) archived the CT images upon admission and the chest radiographs obtained within 24 hs of the CT examinations. Radiographs were available in 72 of 79 patients. The patient outcome was classified in terms of exacerbation during the short-term clinical course; the exacerbated group required a ventilator and was transferred to the intensive care unit in another institution, and the improved group was discharged without being transferred to another institution for ventilator support. The ALI equation is as follows: *ALI = BMI x Alb/NLR*

We also assessed each patient's risk of developing critical illness using an established predictive scoring system reported by Liang *et al* (22). Liang's risk score predictors include abnormality on chest radiography, age, hemoptysis, dyspnea, unconsciousness, number of comorbidities, cancer history, NLR, lactate dehydrogenase, and direct bilirubin (calculator is available in the following website: https://reference.medscape.com/calculator/750/covid-19-critical-illness-prediction-tool-covid-gram).

### Image evaluation

**Figure 1:**
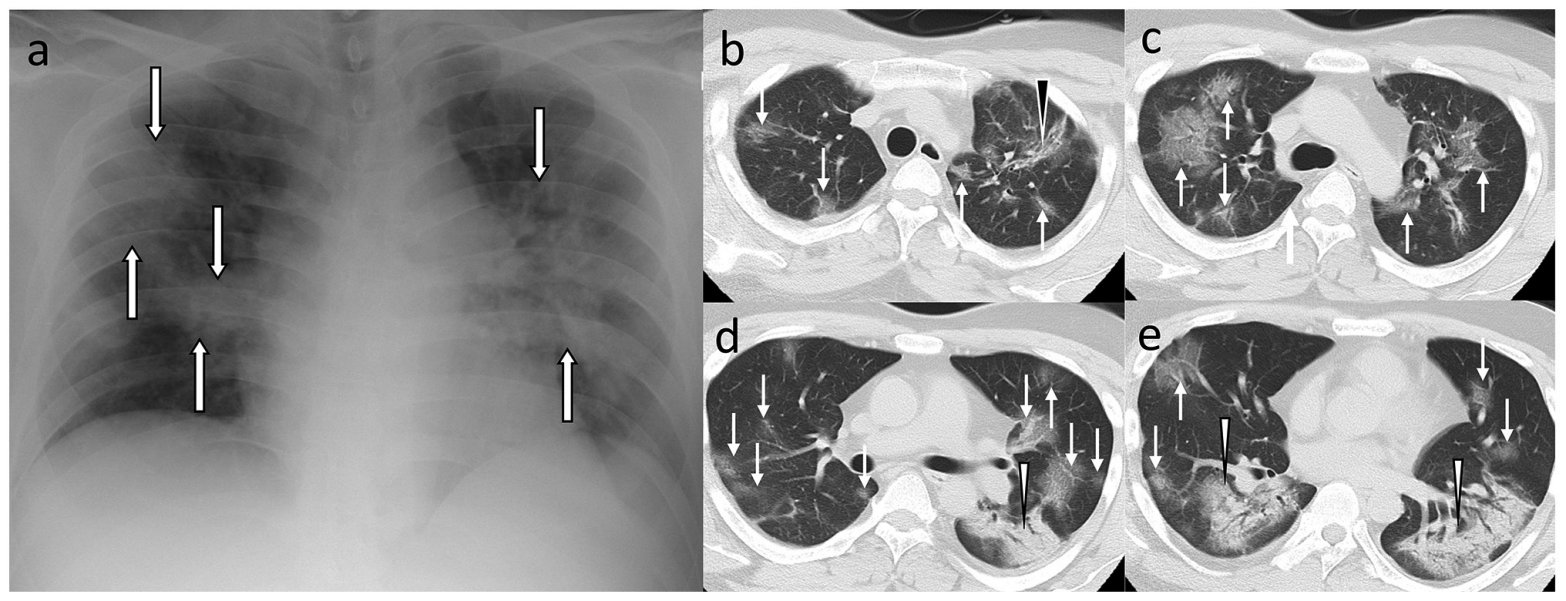
Exacerbation of COVID-19 pneumonia in a 30-year-old man. (a) Chest radiography revealed patchy infiltrations in the bilateral lung (indicated using arrows). (b-e) CT image demonstrated numerous ground-glass opacities (indicated using white arrows) in the whole lung, linear opacity associated with ground-glass opacity [(b) black arrowhead], and consolidation with air bronchogram in the bilateral lower lung lobe [(d and e) white arrowheads]. CT, computed tomography.

A radiologist (A.I., with 12 years of experience in imaging diagnosis) evaluated the chest radiographs (positive or negative for pulmonary opacity). As a semiquantitative approach, another radiologist (H.T., with 7 years of experience in imaging diagnosis) rated the scores using the chest CT score (CCTS; Fig. 1) (23). We used the CCTS as it has been demonstrated to show the best performance among the three semiquantitative CT scoring systems, namely, CCTS (23), total CT score (24), and CT severity score (25,26). The radiologist reviewed the chest CTs with the lung window setting and evaluated the extent of disease involvement using a five-point scale (0: 0%, 1: 0-4%, 2: 5-25%, 3: 26-49%, 4: 50-75%, and 5: 76-100%) for five lung lobes. The patient-level score (0-25) was calculated by summing up the five lobe-level score.

### Statistical analysis

Descriptive statistics demonstrated the frequency (%) or median and interquartile range for each parameter. We employed Fisher's exact test or Mann-Whitney *U* test to compare each parameter between the groups. The combined ALI and radiologic risk prediction model was constructed *via* random forest classification using variables that have significant difference between the improved and exacerbated groups by univariate analysis (age, ALI, severity at admission, chest radiograph abnormality, and CCTS). The accuracy of predicting exacerbation using the ALI, CCTS, and Liang's clinical risk score was evaluated using the receiver-operating characteristic (ROC) curve analysis. A P value <0.05 was considered to indicate a statistically significant difference. All statistical tests were conducted using the R software (version 4.0.2; R Foundation for Statistical Computing, Vienna, Austria).

## Results

Among 79 patients, 72 experienced improvement, and 7 experienced exacerbation after admission. The univariate analysis revealed a significant difference between the improved and exacerbated groups in age (34 vs. 62 years; P=0.032), WBC (median: 4,550 vs. 7,300/µl, P=0.027), NLR (median: 2.2 vs. 7.2, P=0.012), albumin level (median: 4.4 vs. 3.7 mg/dl, P=0.004), ALI (47.6 vs. 13.2; P=0.011), frequency of chest radiograph abnormality (24.7 vs. 83.3%; P<0.001), CCTS (1 vs. 9, P<0.001), Liang score (49.1 vs. 110.6; P<0.001), and severity at admission (rate of severe case: 5.6 vs. 85.7%; P<0.001) (Table I).

During the entire clinical course, 15.2% (12/79) of patients inhaled oxygen, 21.5% (17/79) of patients were administered with systemic steroid therapy, and 15.2% (12/79) of patients were given inhaled steroid therapy. Favipiravir and nafamostat tocilizumab were used in 16.5% (13/79) and 8.8% (7/79) of patients, respectively. Supplemental antibiotics were administered in 8.8% (7/79) of patients (Table I).

**Figure 2:**
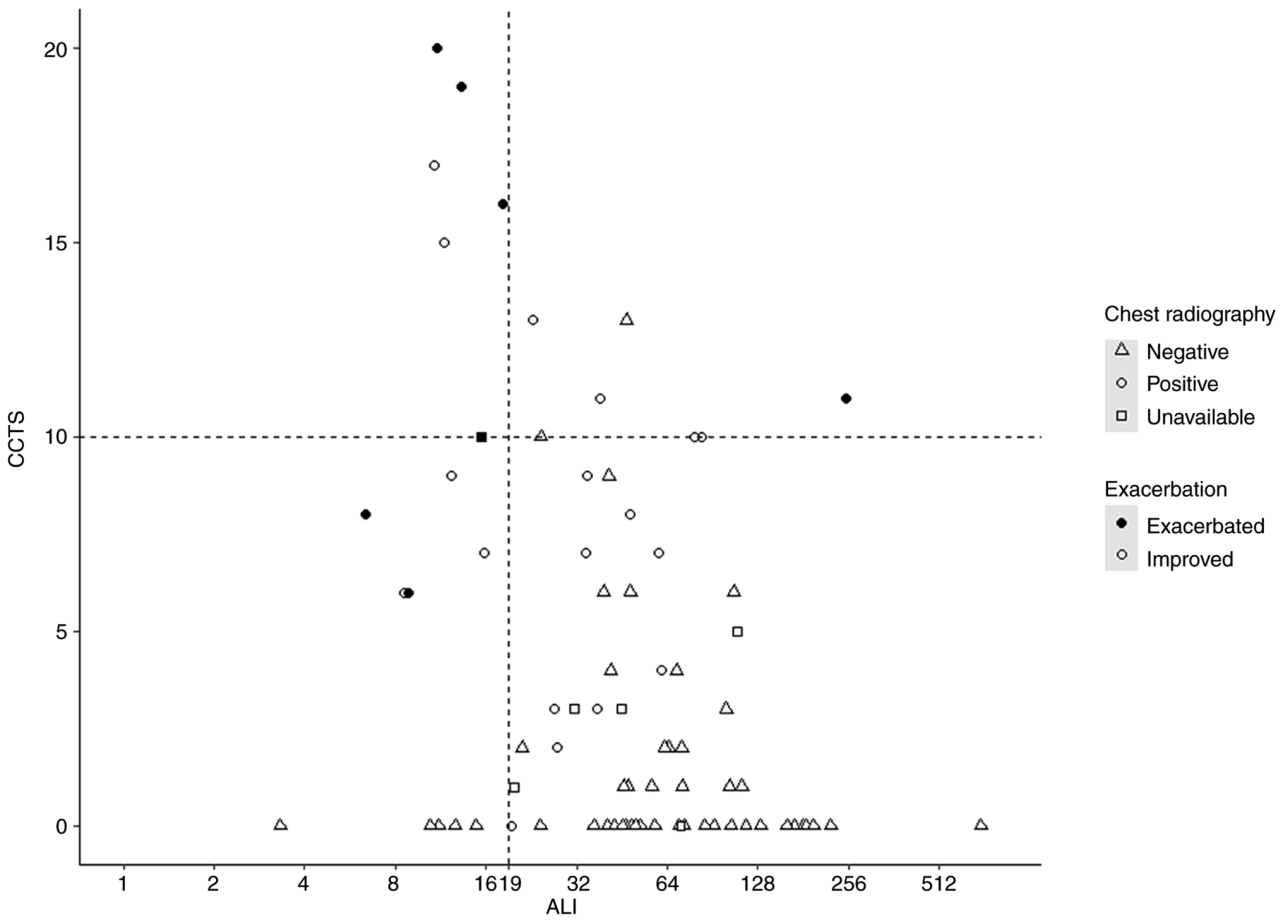
Scatter plot of the relationship between the ALI score and chest CT score. Black circles indicate exacerbated patients, whereas the white circles show the improved patients. The vertical line shows the cutoff value of the ALI score; six of seven exacerbated patients are plotted in the area of ALI <19. The horizontal line indicates the cutoff value of the CCTS; five of seven exacerbated patients were plotted as CCTS ≥10. A total of two patients (body mass index >30 kg/m^2^) with positive chest radiography demonstrated a CCTS ≥10 (triangle). ALI, advanced lung cancer inflammation index; CCTS, chest CT score; CT, computed tomography.

Fig. 2 presents the relationship between the ALI and CCTS, and Tables II and III summarizes the detailed results of the ALI and imaging findings, including CCTS and radiograph. Six of seven exacerbated cases had an ALI less than 19 (Table II), with a BMI ranging from 18.7 to 31.7 kg/m^2^, NLR ranging from 3.5 to 12.7, and albumin ranging from 2.9 to 4.0 g/dl. One exacerbated case had an outlier ALI score of 250.6, with a BMI of 36.6 kg/m^2^, NLR of 0.7, and albumin of 4.7 g/dl (Fig. 1). Among the exacerbated cases, the CCTS ranged from 6 to 20. Among the 72 patients who underwent chest radiography, all patients who experienced exacerbation (n=6) exhibited a positive abnormality on chest radiograph (Table III). Liang's clinical risk score was available in 89.8% (71 of 79) of patients due to the lack of chest radiograph (n=7) and laboratory data (n=1).

**Figure 3:**
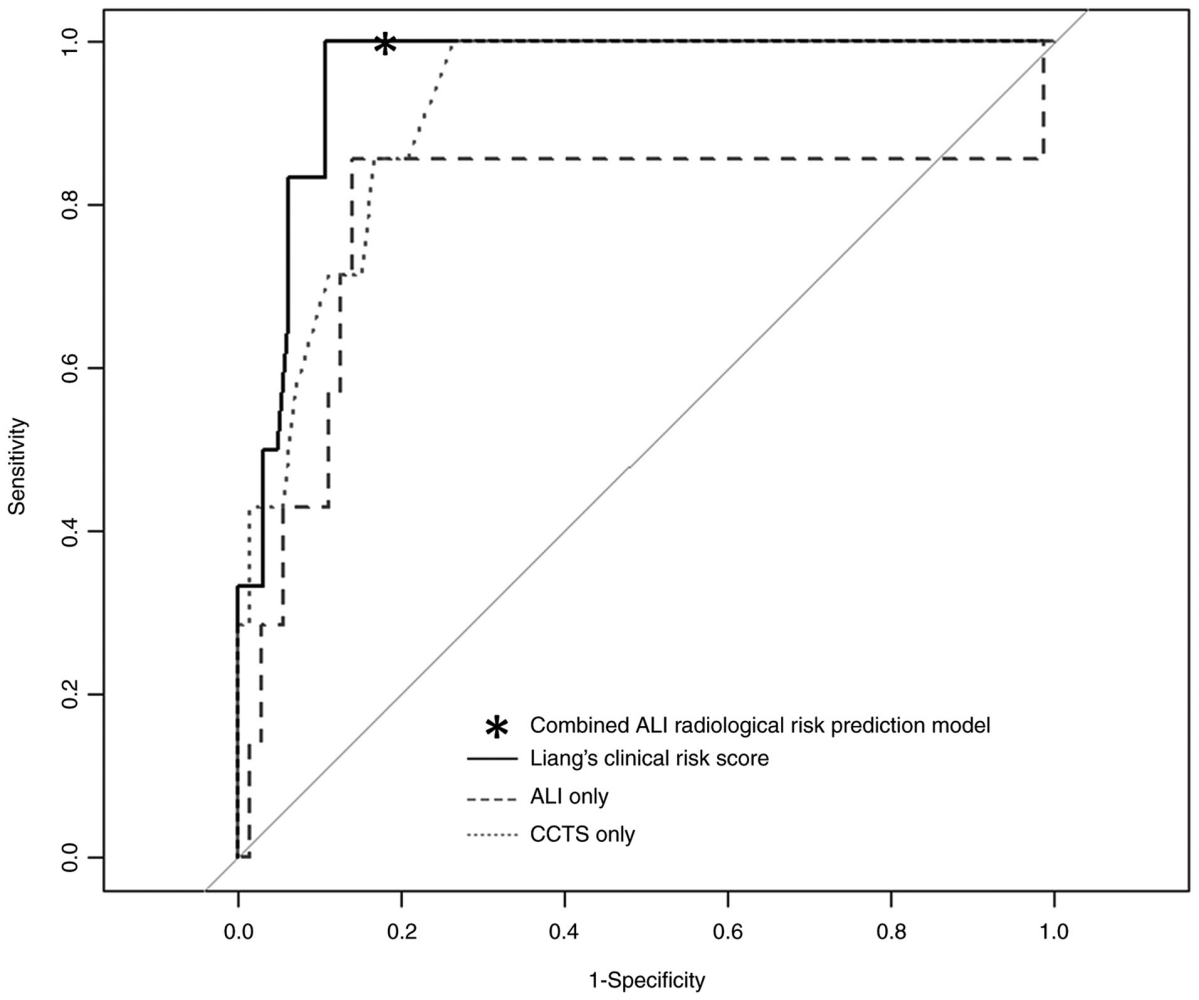
Receiver-operating characteristic curve for predicting exacerbation. Areas under the curve were 0.79 (95% CI, 0.53-1.00) in ALI, 0.92 (95% CI, 0.84-0.92) in CCTS and 0.96 (95% CI, 0.91-1.00) in Liang's clinical risk score. With a cutoff value determined by the Youden index, Liang's clinical risk score had a sensitivity of 1.00 and specificity of 0.89. The combined ALI and radiologic risk prediction model exhibited a sensitivity of 1.00 and specificity of 0.81. ALI, advanced lung cancer inflammation index; CCTS, chest computed tomography score; CI, confidence interval.

For the accuracy of predicting exacerbation, the ROC analysis demonstrated AUCs of 0.79, 0.92, and 0.96 for the ALI, CCTS, and Liang's clinical risk scores, respectively (Fig. 3). With a cutoff value of 19, the ALI alone had a sensitivity of 0.86 and specificity of 0.86. With a cutoff value of 10, the CCTS alone had a sensitivity of 0.72 and a specificity of 0.89. With a cutoff value of 88.4 calculated using the Youden index, Liang's clinical risk score had a sensitivity of 1.00 and a specificity of 0.89.

**Figure 4:**
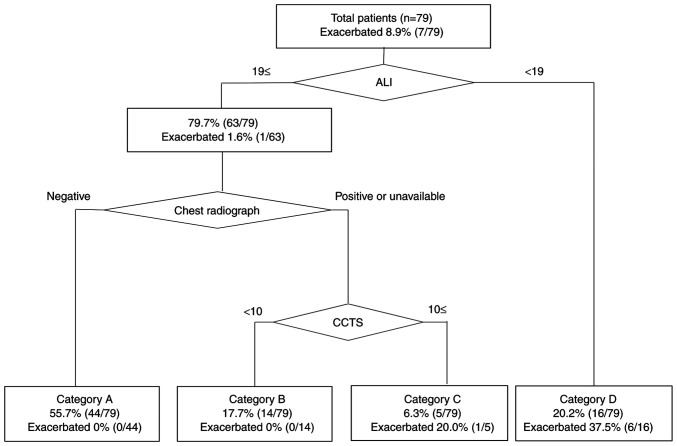
Combined ALI and radiologic risk prediction model. Upper number indicates the percentage of patients in each category, and the lower number indicates the percentage of exacerbated patients in each category. All patients (73.4%, 56/79) were improved in the cluster of both ALI ≥19 and CCTS <10 (categories A and B). In 6.3% (5/79) of patients presenting with both ALI ≥19 and CCTS ≥10, 14.3% (1/5) of the patients were exacerbated (category C). In 20.2% (16/79) of patients with an ALI of <19, 37.5% (6/16) were exacerbated (category D). ALI, advanced lung cancer inflammation index; CCTS, chest computed tomography score.

The combined ALI and radiologic risk prediction model (Fig. 4) was developed using the ALI, chest radiograph abnormality, and CCTS as the first-, second-, and third-step significant classifiers, respectively. The result of random forest classification itself adopted only two classifiers (ALI and CCTS), and therefore we manually inserted chest radiograph as the second classifier to fit our clinical practice. This combined model categorized patients into four groups: category A (ALI ≥19 and negative chest radiograph abnormality: 55.7% [44/79]), category B (ALI ≥19 and positive or nonavailable chest radiograph and CCTS <10: 17.7% [14/79]), category C (ALI ≥19 and positive or nonavailable chest radiograph and CCTS ≥10: 6.3% [5/79]), and category D (ALI <19: 20.2% [16/79]). Exacerbation was observed in one patient in category C and in six patients in category D. The combined ALI and radiologic risk prediction model had a sensitivity of 1.00 and a specificity of 0.81 when categories A and B were considered as negative and categories C and D as positive.

## Discussion

Our findings indicate that the ALI score was significantly higher in the improved group (47.6) than in the exacerbated group (13.2; P=0.011), and patients with a lower ALI score had a greater probability of CT or chest radiograph abnormalities. The ALI alone and CCTS alone had a modest AUC for predicting exacerbation of COVID-19 (0.79 and 0.92). The combined ALI and radiologic risk prediction model had decent sensitivity (1.00) and specificity (0.81), which was almost similar to the established clinical risk score (Liang's clinical risk score: sensitivity of 1.00 and specificity of 0.89).

Our combined ALI and radiologic risk prediction model adopted an ALI threshold of 19, which was included within the range of the previously documented ALI thresholds (15.5-43.5) for other diseases (12,16,17,27-33). The ALI is directly proportional to the BMI and albumin and inversely proportional to the NLR. In our study, exacerbated cases exhibited a significantly higher NLR (7.2 vs. 2.2) and lower albumin (3.7 vs. 4.4) compared with improved cases, which should result in a significantly lower ALI in exacerbated cases. Six of the seven exacerbated cases had an ALI <19. One exacerbated case had an outlier ALI of 250.6, which could be explained by obesity (i.e., high BMI of 36.6) and low NLR of 0.7. This case suggested that the ALI might not necessarily reflect the actual risk in patients with high BMI. If we assume that there are two patients with similar values of albumin and NLR, a patient with a lower BMI should be categorized as a higher risk (i.e., lower ALI). This could be true in the setting of evaluating patients with cachexia but not for patients with obesity (high BMI), which is known to be an important risk factor for COVID-19 exacerbation (20). Thus, the ALI may underestimate the potential risk of COVID-19 exacerbation in obese patients. The modest accuracy of the ALI in predicting COVID-19 exacerbation in our study might be due to the lower proportion of obese patients in our patient cohort (BMI >30: 14% [11/78]) compared with the previous study (34,35). Another potential factor that could influence the accuracy of ALI risk prediction is NLR. The ALI is inversely proportional to NLR, and therefore, patients with a low NLR value could have an extraordinarily high ALI value. The one exacerbated case with outlier ALI had a lower NLR (0.7) compared with other exacerbated cases (3.5-12.7). Therefore, the application of the ALI should be avoided in patients with neutropenia.

The current guidelines do not recommend routine chest radiography and CT for all COVID-19 patients (36,37). The avoidance of unnecessary imaging examinations is important for the reduction of radiation exposure for patients, viral exposure for medical staff, and medical costs (38). Our combined ALI and radiologic risk prediction model helps avoid performing unnecessary imaging for patients with a low risk of exacerbation. In our study cohort, 55.7% (44/79) of patients were categorized as category A, and they can waive CT scans based on the negativity of chest radiograph and ALI ≥19. CT scan was suggested only for patients with a positive radiograph, and a positive chest radiograph in patients with COVID-19 was correlated with higher CCTS scores. Of the 48 patients in our study, 2 with a negative radiograph (4.2%) had a CCTS ≥10, whereas 10 of the 24 patients with a positive radiograph (42.0%) had a CCTS ≥10. We must be aware of the probability of a false-negative result on radiography. In our study, the BMI of the two patients with a negative radiograph and a CCTS ≥10 was more than 30 kg/m^2^. We considered that a large body size could degrade the image quality of radiograph, which could result in false-negative results.

Despite its high accuracy in predicting COVID-19 exacerbation, the major drawback of Liang's clinical risk score is that it requires us to input a fairly large number of clinical data, including patient's history, chest radiograph, and lab values (lactate dehydrogenase and direct bilirubin) to obtain the estimated risk results. On the contrary, the ALI can be calculated using only the BMI, albumin level, and NLR and is therefore available in home medical care settings or primary care clinics. We can now expect the application of our combined ALI and radiologic risk prediction model in the primary care facility. The patients in category D (i.e., ALI <19) are at a high risk and therefore should be evaluated further, treated intensively, or referred to a hospital. The patients in other categories (i.e., ALI ≥19) should be evaluated with additional chest radiograph at primary care facilities. In our model, negativity of chest radiograph could waive CT scans in a large amount of patients with low risk of exacerbation (category A). However, patients with a positive chest radiograph should be subsequently assessed with CT scans to distinguish those at a high risk (category C) who should be treated intensively or referred to a hospital from those at a low risk (category B) who may be followed-up at a primary care facility. We adopted a CCTS threshold of 10 to distinguish between groups B and C, which is slightly higher than the previously reported threshold (CCTS of 7) used to identify the critical disease of COVID-19 pneumonia upon admission with a sensitivity of 0.80 and a specificity of 0.83(23). This means that our model's threshold has presumably higher specificity and lower sensitivity for distinguishing group C from group B. This is reasonable because we used the CCTS as the third classifier, and most low risk patients were already classified into group A using the ALI and radiograph. Our threshold was lower than another threshold (CCTS of 18) used to predict mortality in a short-term follow-up (39), indicating that our model could include less severe cases in group C than those at a high risk of short-term death. As such, our combined ALI and radiologic risk prediction model could be helpful in the rapid decision making for patient triage during the COVID-19 pandemic, when medical resources are limited.

This study has several limitations. First, the study design was retrospective in nature with a relatively small number of patients experiencing exacerbation. The survival rate was not determined because the patients with exacerbation were transferred to the tertiary referral institution equipped with an intensive care unit. Second, we did not conduct an external validation of our model. Third, our patient cohort consisted of admitted patients and did not include outpatients. Further analysis is necessary to apply our model in an external patient cohort that includes both inpatients and outpatients. Forth, we didn't include COVID-19 negative patients in our study cohort. The purpose of our study is to evaluate the usefulness of applying our combined model to patients with known diagnosis of COVID-19 confirmed by polymerase chain reaction test, and we didn't intend to apply this model to patients without COVID-19 infection. Therefore, no control group enrollment in this study does not affect our result. Finally, the patients were enrolled in this study before the vaccine was released. The COVID-19 vaccine obviously prevents severe disease and exacerbation (40); therefore, it is unknown if the results can be similarly applied to the vaccinated population.

In conclusion, the ALI could be applicable in evaluating the risk of COVID-19 infection. Patients with COVID-19 infection who have a lower ALI score tend to have a higher probability of CT or chest radiograph abnormalities. The ALI alone and CCTS alone modestly predict the exacerbation of COVID-19, and the combined ALI and radiologic risk prediction model exhibit decent sensitivity and specificity. However, prediction using the ALI may not be accurate in patients with obesity or neutropenia.

## Figures and Tables

**Table I:** Patient characteristics and results of the univariate analysis.

Variables	Total (n=79)	Improved group (n=72)	Exacerbated group (n=7)	P-value
Patient demographics				
Median age, years (IQR)	35 (26, 58)	34 (25, 56)	62 (42, 76)	0.032^a^
Female (%)	34 (43.0)	33 (45.8)	1 (14.3)	0.229
Body weight, kg (IQR)	62.0 (53.6, 77.7)	61.9 (53.4, 77.2)	63.0 (62.1, 89.3)	0.259
Height, cm (IQR)	163.8 (159.1, 170.2)	163.7 (158.7, 170.9)	169.0 (163.0, 169.4)	0.227
Body mass index (IQR)	23.4 (21.4, 27.0)	23.3 (21.2, 26.8)	24.1 (22.8, 28.7)	0.422
Laboratory results				
WBC (/µl), (IQR)	4,700 (3,700, 6,350)	4,550 (3,700, 6,025)	7,300 (4,800, 8,350)	0.027^a^
Neut/Lymph ratio (IQR)	2.30 (1.40, 4.00)	2.20 (1.38, 3.70)	7.20 (4.95, 9.40)	0.012^a^
Albumin, g/dl (IQR)	4.40 (4.00, 4.60)	4.40 (4.10, 4.60)	3.70 (3.65, 3.90)	0.004^a^
Scoring				
ALI (IQR)	46.5 (23.6, 75.9)	47.6 (27.3, 80.0)	13.2 (9.9, 16.9)	0.011^a^
Liang score (IQR)	51.5 (32.0, 81.4)	49.1 (31.7, 71.6)	110.6 (102.2, 121.9)	<0.001
Imaging examination				
Chest radiograph abnormality (%)^b^	24 (32.9)	18 (24.7)	6 (83.3)	<0.001^a^
Chest CT score (IQR)	2 (0, 7)	1 (0, 6)	9 (9, 17.5)	<0.001^a^
Severity at admission				
Mild	69 (87.3)	68 (94.4)	1 (14.3)	<0.001^a^
Severe	10 (12.7)	4 (5.6)	6 (85.7)	
Treatment, n (%)				
Oxygen inhalation	12 (15.2)	6 (8.3)	6 (85.7)	<0.001^a^
Active treatment	30 (40.0)	25 (34.7)	5 (71.4)	1.000
Favipiravir	13 (16.5)	10 (13.9)	3 (42.9)	0.083^a^
Nafamostat	7 (8.9)	5 (6.9)	2 (28.6)	0.115
Systemic steroid	17 (21.5)	13 (18.1)	4 (57.1)	0.035^a^
Inhaled steroid	12 (15.2)	12 (16.7)	0 (0)	0.587
Anticoagulants	2 (2.5)	1 (1.4)	1 (14.3)	0.170
Antibiotics	7 (8.9)	3 (4.2)	4 (57.1)	<0.001^a^

^a^P<0.05;

^b^Chest radiograph was available in 73/79 patients. ALI, advanced lung cancer inflammation index; CCTS, chest CT score; IQR, interquartile range; CT, computed typography.

**Table II:** Relationship between the ALI and chest CT score.

ALI (n=79)	ALI ≥19 (n=63)	ALI <19 (n=16)	P-value
Exacerbation (%)	1 (15.9)	6 (37.5)	<0.001^a^
CCTS (IQR)	1 (0, 4.5)	7.5 (0, 15.25)	0.014^a^
CCTS ≥10 (%)	7 (11.1)	6 (37.5)	0.021^a^

^a^P<0.05. ALI, advanced lung cancer inflammation index; CT, computed tomography; CCTS, chest CT score.

**Table III:** Relationship between chest radiograph abnormality and other parameters.

Chest radiograph (n=72)	Negative (n=48)	Positive (n=24)	P-value
Exacerbation (%)	0 (0)	6 (25.0)	<0.001^a^
ALI (IQR)	56.9 (41.0, 103.1)	25.1 (12.1, 40.8)	<0.001^a^
ALI <19 (%)	5 (10.4)	10 (41.7)	0.004^a^
CCTS (IQR)	0 (0, 2)	8.5 (6, 11.5)	<0.001^a^
CCTS ≥10 (%)	2 (4.2)	10 (41.7)	<0.001^a^

^a^P<0.05. ALI, advanced lung cancer inflammation index; CT, computed tomography; CCTS, chest CT score.

## Data Availability

The datasets used and/or analyzed during the current study are available from the corresponding author on reasonable request.

## Authors' information

Dr Akitoshi Inoue (ORCID: 0000-0002-8610-2571); Dr Hiroaki Takahashi (ORCID: 0000-0002-8731-0081); Dr Yoichiro Hamamoto (ORCID: 0000-0002-2165-9475).
